# Immunomics-Guided Antigen Discovery for Praziquantel-Induced Vaccination in Urogenital Human Schistosomiasis

**DOI:** 10.3389/fimmu.2021.663041

**Published:** 2021-05-25

**Authors:** Mark S. Pearson, Bemnet A. Tedla, Luke Becker, Rie Nakajima, Al Jasinskas, Takafira Mduluza, Francisca Mutapi, Claude Oeuvray, Beatrice Greco, Javier Sotillo, Philip L. Felgner, Alex Loukas

**Affiliations:** ^1^ Centre for Molecular Therapeutics, Australian Institute of Tropical Health and Medicine, James Cook University, Cairns, QLD, Australia; ^2^ Vaccine Research and Development Center, Department of Physiology and Biophysics, University of California Irvine, Irvine, CA, United States; ^3^ Department of Biotechnology and Biochemistry, University of Zimbabwe, Harare, Zimbabwe; ^4^ TIBA Partnership, NIHR Global Health Research Unit Tackling Infections to Benefit Africa (TIBA) at the University of Edinburgh based in Harare (TIBA Zimbabwe), Harare, Zimbabwe; ^5^ Institute of Immunology and infection Research, Ashworth Laboratories, Edinburgh, United Kingdom; ^6^ TIBA Partnership, NIHR Global Health Research Unit Tackling Infections to Benefit Africa (TIBA) at the University of Edinburgh, Edinburgh, United Kingdom; ^7^ Global Health Institute of Merck, Ares Trading S.A., a subsidiary of Merck KGaA (Darmstadt, Germany), Eysins, Switzerland; ^8^ Parasitology Reference and Research Laboratory, Centro Nacional de Microbiología, Instituto de Salud Carlos III, Madrid, Spain

**Keywords:** cystatin, praziquantel, proteome microarray, urogenital schistosomiasis, vaccine, immunomics

## Abstract

Despite the enormous morbidity attributed to schistosomiasis, there is still no vaccine to combat the disease for the hundreds of millions of infected people. The anthelmintic drug, praziquantel, is the mainstay treatment option, although its molecular mechanism of action remains poorly defined. Praziquantel treatment damages the outermost surface of the parasite, the tegument, liberating surface antigens from dying worms that invoke a robust immune response which in some subjects results in immunologic resistance to reinfection. Herein we term this phenomenon Drug-Induced Vaccination (DIV). To identify the antigenic targets of DIV antibodies in urogenital schistosomiasis, we constructed a recombinant proteome array consisting of approximately 1,000 proteins informed by various secretome datasets including validated proteomes and bioinformatic predictions. Arrays were screened with sera from human subjects treated with praziquantel and shown 18 months later to be either reinfected (chronically infected subjects, CI) or resistant to reinfection (DIV). IgG responses to numerous antigens were significantly elevated in DIV compared to CI subjects, and indeed IgG responses to some antigens were completely undetectable in CI subjects but robustly recognized by DIV subjects. One antigen in particular, a cystatin cysteine protease inhibitor stood out as a unique target of DIV IgG, so recombinant cystatin was produced, and its vaccine efficacy assessed in a heterologous *Schistosoma mansoni* mouse challenge model. While there was no significant impact of vaccination with adjuvanted cystatin on adult worm numbers, highly significant reductions in liver egg burdens (45-55%, *P*<0.0001) and intestinal egg burdens (50-54%, *P*<0.0003) were achieved in mice vaccinated with cystatin in two independent trials. This study has revealed numerous antigens that are targets of DIV antibodies in urogenital schistosomiasis and offer promise as subunit vaccine targets for a drug-linked vaccination approach to controlling schistosomiasis.

## Introduction

Schistosomiasis is a chronic, often debilitating, parasitic disease afflicting over 250 million people worldwide and is responsible for the loss of approximately 1.9 million disability adjusted life years (DALYs) (1). Three schistosome species - *Schistosoma mansoni, S. haematobium* and *S. japonicum* - account for almost all human infections. Adult *S. mansoni* and *S. japonicum* live in the portal and mesenteric veins while *S. haematobium* lives in the veins of the bladder, where the male and female flukes pair and survive for many years, producing hundreds of fertilized eggs per day. Severe morbidity results from host immune responses to eggs in tissues, and includes periportal fibrosis, portal hypertension, urinary obstruction, and bladder carcinoma. Currently, chemotherapy with praziquantel (PZQ) is the preferred treatment for schistosomiasis, although control programs based on mass drug administration/preventative chemotherapy are complicated by rapid and frequent re-infection and the difficulties and expense of maintaining these programs over a long term (2).

PZQ is widely used to treat human schistosome infections and has two main effects on the parasites – rapid paralysis and tegument damage. Experimental work in mice has demonstrated that PZQ treatment exposes tegumental (surface membrane) antigens (3). In addition, adult worms are believed to suppress schistosome-specific immune responses so that worm death results in increased responsiveness to schistosome antigens (4). The interaction of PZQ with schistosomes results in a quantitative and qualitative alteration of host–parasite-specific immune responses. Early studies reported modifications in the cell proliferative responses (4) and in the levels and types of antibody (5, 6) and cytokine responses (7) following treatment. While this infection and treatment approach to inducing immunologic resistance to reinfection has been well articulated (8), the antigen targets of protective immune responses in human subjects is poorly understood. We refer to this phenomenon herein as “Drug Induced Vaccination (DIV)”. Very few schistosomiasis vaccine candidates have progressed towards commercial development (9), and none of those antigens in development were identified using a DIV-based strategy. Innovative approaches are therefore needed to identify the protective schistosome proteins that are the target of DIV, and develop them into a subunit vaccine.

An “immunome” can be defined as the entire set of antigens or epitopes that interface with a host immune system. Recent advances in high order multiplexing, often referred to as megaplexing, provide a practical, high throughput, and affordable approach to estimating the immunomic profiles of a human or animal to a pathogen (10, 11). The advantage of the systems biology approach is that it aims to look at the totality of the humoral immune response to an infectious disease, attempting to be as complete and accurate as possible (12). It takes advantage of genomics to design primers to amplify and clone each annotated gene from dozens of infectious agents, enabling the expression of thousands of proteins on a genome-wide scale. This approach permits investigators to assess the repertoire of antibodies and can be used to perform large-scale analyses to determine whole profiles of reactive antigens and patient-to-patient and species-dependent differences in the response to infection while empowering statistical conclusions. We applied protein array technology for the first time to study the immune response to a eukaryotic pathogen (*S. mansoni*), with the creation of the first schistosome protein array which contained a small representative subset of *S. mansoni* and *S. japonicum* surface proteins (10). A subsequent study screened this array with a small number of serum samples from human subjects infected with *S. haematobium* who had been treated with a single dose of 40 mg/kg PZQ and either became reinfected or developed DIV (13). While a valuable study that revealed new and previously known vaccine candidate antigens, major limitations were the small number of arrayed antigens and the cross-species nature of the study – i.e., select *S. mansoni* and *S. japonicum* arrayed antigens probed with sera from *S. haematobium* infected individuals.

Herein we describe the construction of a *S. haematobium* protein microarray consisting of almost 1,000 feature proteins (selected by a multi-omics approach consisting of proteomics, transcriptomics, and bioinformatics), probing of that array to identify targets of DIV and validation of the vaccine efficacy of the top-ranking antigen target in a murine model of schistosomiasis.

## Materials and Methods

### Ethical Statement, Study Design and Cohorts

Ethical approval for sample collection from human subjects in Zimbabwe was obtained by the Medical Research Council of Zimbabwe. The demographics of the study population has been described in depth elsewhere (13), but briefly, study participants were residents of a *S. haematobium*-endemic rural village in Murewa in the Mashonaland East Province of Zimbabwe. The village has little or no infection with soil-transmitted helminths and a low *S. mansoni* prevalence (<2%). Serum samples were provided from a cohort of *S. haematobium*-infected individuals (*n* = 106) aged 5–14 years who had never been treated with PZQ prior to this study and were free from co-infection with other helminths, *Plasmodium*, and HIV. At the start of the study (baseline), subjects who were positive for *S. haematobium* eggs (at least one egg found in at least one of three urine samples, each collected on a separate day) following urinalysis were treated with PZQ by weight (40 mg/kg) and then assessed by urinalysis at 6 weeks to confirm clearance of the infection (no eggs found in any of three urine samples, each collected on a separate day). Individuals were followed for 18 months and maintained regular water contact throughout this period. Subjects were assessed for infectivity with *S. haematobium* at 6 months and at the end of the study. Individuals who were egg-positive at 18 months post-treatment (*n* = 32) were deemed CI and those who were egg-negative (*n* = 74) were categorized as DIV. Serum samples were obtained from both 0- and 18-month timepoints. For this study, we selected a subset of subjects as follows: CI subjects that had the highest post-treatment egg burdens (eggs/10 ml 10–104; *n* = 13) and DIV subjects that had some of the highest egg burdens at baseline (eggs/10 ml 44–743; *n* = 16), reasoning that these individuals represented extremities of the DIV and CI spectrums and therefore would maximize the likelihood of identifying differences in antibody signatures between CIs and DIVs.

On collection, all serum and urine samples were stratified based on egg burden as determined by microscopy analysis of urine samples (high, ≥50 eggs per 10 ml urine; or light, 1-49 eggs per 10 ml urine).

### *S. haematobium* Protein Array Feature Selection and Construction

Protein selection criteria for inclusion in the *S. haematobium* protein array was the presence of the protein in surface or secreted proteome data sets. Accordingly, proteins in adult *S. haematobium* tegument, adult soluble excretory/secretory products (ES) (14) and extracellular vesicles (EVs) (15), egg ES and soluble egg antigen (SEA) (14), and *S. haematobium* orthologues of *S. mansoni* schistosomula tegument proteins (16) (650 proteins in total) were selected for printing on the array. The remaining ~350 proteins were *S. haematobium* orthologues of proteins printed on a *S. mansoni* second generation array (17). The list of sequences selected for inclusion in the array can be accessed through Mendeley Data at the following link: doi:10.17632/6c4bzj9nzr.1. Open reading frames with predicted signal peptides removed were flanked by 20 bp sequences corresponding to pXI recombination sites (18) were codon optimised for expression in *E. coli* and commercially synthesised and cloned in pUC57 by either Twist Bioscience (sequences < 1.8 kb) or ProteinCT (sequences > 1.8 kb). Synthesised genes were amplified by PCR using oligonucleotides corresponding to the pXI recombination sites and cloned into pXI, in-frame with the sequences encoding the 5’ HA and 3’ HIS tags using *in vivo* recombination (19). Plasmid minipreps were produced and proteins encoded by each purified plasmid were expressed *in vitro* (RTS 100 *E. coli* HY kit – 5 Prime, MD, USA) according to the manufacturer’s instructions and printed onto 8-pad nitrocellulose-coated AVID glass slides (Grace Biolabs, Bend, OR, USA) with an Omnigrid 100 microarray printer (Genomic Solutions, Ann Arbor, MI, USA). Vector without insert was similarly produced and printed (in multiple locations) as a negative control. Multiple empty spots were left on each pad to serve as background controls. Purified human IgG, anti-human IgG and schistosome extracts (*S. haematobium* adult- and egg-stage ES products, soluble egg antigen and adult-stage tritonX-100-soluble extract) were also printed as positive controls. Expression quality control was assessed by detection of N-terminal HA and C-terminal HIS tags as previously described (18).

### Probing of Protein Arrays With Human Sera

IgG responses to antigens on the array were determined by probing with human serum (1:50 in array blocking buffer/10% *E. coli* lysate) as previously described (18) with the exception that anti-human IgG-Qdot (1:100 in array blocking buffer) was used as the secondary antibody.

### Data Analysis and Bioinformatics

After subtracting the background from each slide, the signal intensity (SI) of each spot was corrected using the “group average” method, where the mean SI of the negative control (empty vector) spots were subtracted from the SI of each protein spot. Corrected SIs were transformed using variance stabilizing normalization (vsn) in GMine (http://cgenome.net/gmine) using the VSN Bioconductor package (20). A reactivity cut-off of 543 (calculated as the mean plus 1.5 standard deviations (SD) of the SI of the negative control spots) was used. Significance differences between antibody responses in DIV versus CI groups were determined by Student’s t-test and p values adjusted for multiple statistical comparisons.

### Recombinant Protein Production in *E. coli*

The top ranked IgG response in terms of highest-fold change in SI between DIV and CI subjects targeted MS3_01693, a cystatin family member. The *S. mansoni* homolog of MS3_01693 (smp_034420) was selected for recombinant expression and validation in our vaccine model (*S. mansoni*) as numbers of *S. haematobium* cercariae were insufficient to perform appropriately powered vaccination trials using an *S. haematobium* model of infection. The ORF was synthesized by Genscript and contained flanking *Nde*I and *Xho*I sites to accommodate cloning into the pET41a expression vector (Novagen) such that the N-terminal GST tag was removed but the C-terminal 6-His tag was retained. Protein expression was induced for 24 h in *E. coli* BL21(DE3) by addition of 1 mM isopropyl beta-D-thiogalactopyranoside (IPTG) using standard methods. Recombinant MS3_01693 required 8M urea to solubilize and was prepared and purified using a method described by us elsewhere (21). The final concentration of purified recombinant protein was adjusted to 1 mg/ml and aliquots were stored at -80°C.

### Vaccine Formulation and Immunization Regimen

Vaccine experiments were performed using an *S. mansoni* challenge model as recently described by us (21). Briefly, two groups of 10 BALB/c mice (6–8 weeks) were immunized intraperitoneally on day 1 with either recombinant *S. mansoni* smp_034420 (cystatin) or recombinant *E. coli* thioredoxin (TrX) expressed and purified in identical fashion as an irrelevant control protein (50 μg/mouse). Proteins were formulated with an equal volume of Imject alum adjuvant (Thermofisher) and 5 μg of CpG ODN1826 (InvivoGen). Two booster immunizations (for a total of three immunizations) were administered on days 15 and 29 and each mouse was challenged by tail penetration with 120 *S. mansoni* cercariae on day 43. Blood was collected by tail bleed at day 42 to determine pre-challenge antibody titres. Two independent trials were performed.

### Necropsy and Estimation of Parasite Burden

Necropsy and quantification of parasite burdens was performed as described elsewhere (21). Mice were necropsied at day 91 (7 weeks post-infection) and blood was collected by cardiac puncture. Adult *S. mansoni* flukes were harvested by vascular perfusion and counted. Livers were removed, weighed, and digested, and schistosome eggs were concentrated by centrifugation at 1,000 *g* for 10 min and re-suspended in 10% formalin. The number of eggs in a 5 μL aliquot was counted in triplicate and the number of eggs per gram (EPG) of liver tissue was calculated. Small intestines were removed and cleaned of debris before being weighed and digested as per the livers. Eggs were similarly concentrated and counted to calculate intestinal EPG.

### Statistics

All statistics were performed using GraphPad Prism 9.0. The reductions in worm and egg numbers were analysed using a Student’s *t*-test and results were expressed as the mean ± standard error of the mean. For antibody titers, the reactivity cut-off values were determined as the mean + 3SD of the values obtained with naive serum.

## Results

### *S. haematobium* Secreted Proteome Array

**Figure 1:**
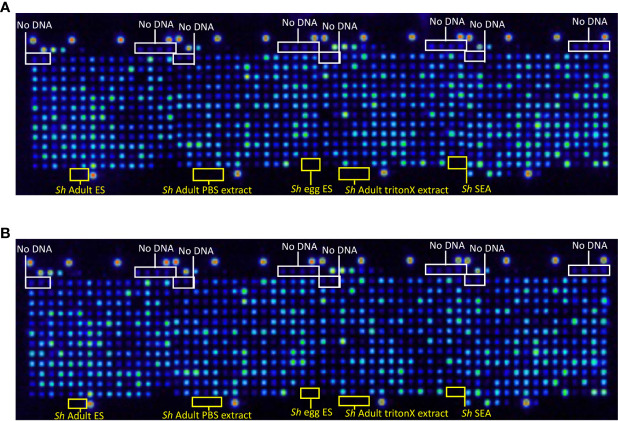
Quality control probe of the *S. haematobium* protein microarray. Arrays were probed with **(A)** anti-hemagglutinin (HA) antibody to detect expression of the N-terminal HA tag and determine the percentage of protein expression from all arrayed antigens, and **(B)** anti-6-HIS antibody to detect expression of the C-terminal 6-HIS tag and determine the percentage of full-length protein expression from all arrayed antigens. No DNA refers to control spots where plasmid DNA (encoding genes to be expressed) is omitted from the reverse transcription-translational products printed at those locations. Sh Adult ES, *S. haematobium* adult fluke Excretory/Secretory products; Sh Adult PBS extract, *S. haematobium* adult fluke PBS-soluble somatic extract; Sh egg ES, *S. haematobium* egg ES products; Sh Adult triton extract, *S. haematobium* adult fluke TritonX-114-soluble extract; Sh SEA, *S. haematobium* soluble egg antigen.

Proteome arrays were probed with monoclonal antibodies raised to the N-terminal HA tag to assess the percentage of proteins expressed and C-terminal His tag to determine the percentage of full-length protein expression from all 992 arrayed antigens. In total, 929 proteins were expressed (**Figure 1A**), and 863 (93%) of those were full-length (**Figure 1B**). Eighty-nine of the proteins selected for printing contained a predicted signal peptide (**Table S1**). We expect the true number to be higher than this due to the draft nature of the genome and consequently incomplete ORFs.

### Immunoreactivity of Arrayed Proteins

**Figure 2:**
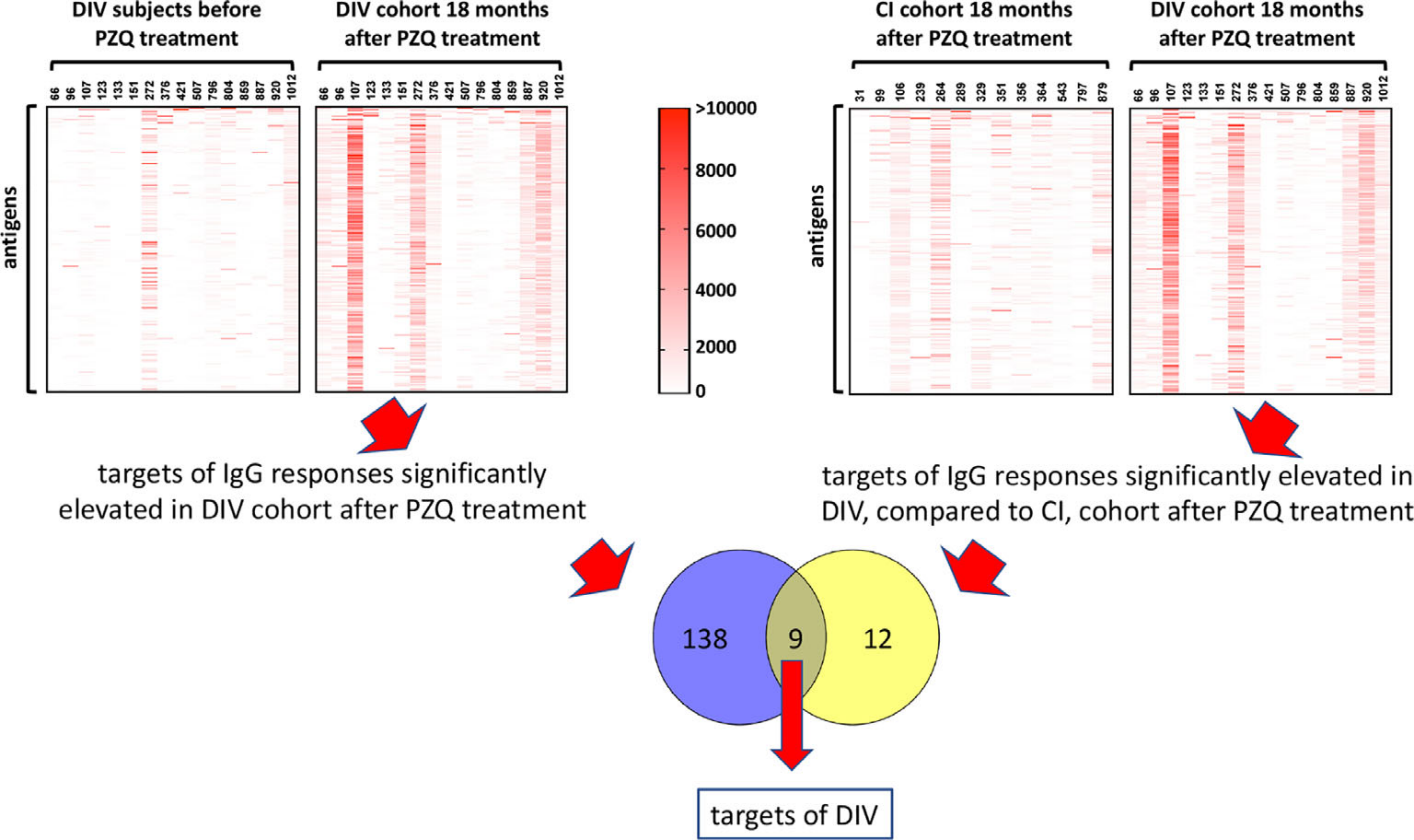
Approach to identification of antigenic targets of DIV. Antigens which were targets of IgG responses that were significantly elevated 18 months after PZQ treatment compared to before treatment in DIV subjects (1, upper left panel), and significantly elevated in DIV subjects compared to CI subjects 18 months after PZQ treatment (2, upper right panel) were considered potential targets of DIV. The Venn diagram shows nine antigens that were shared between the two datasets.

**Figure 3:**
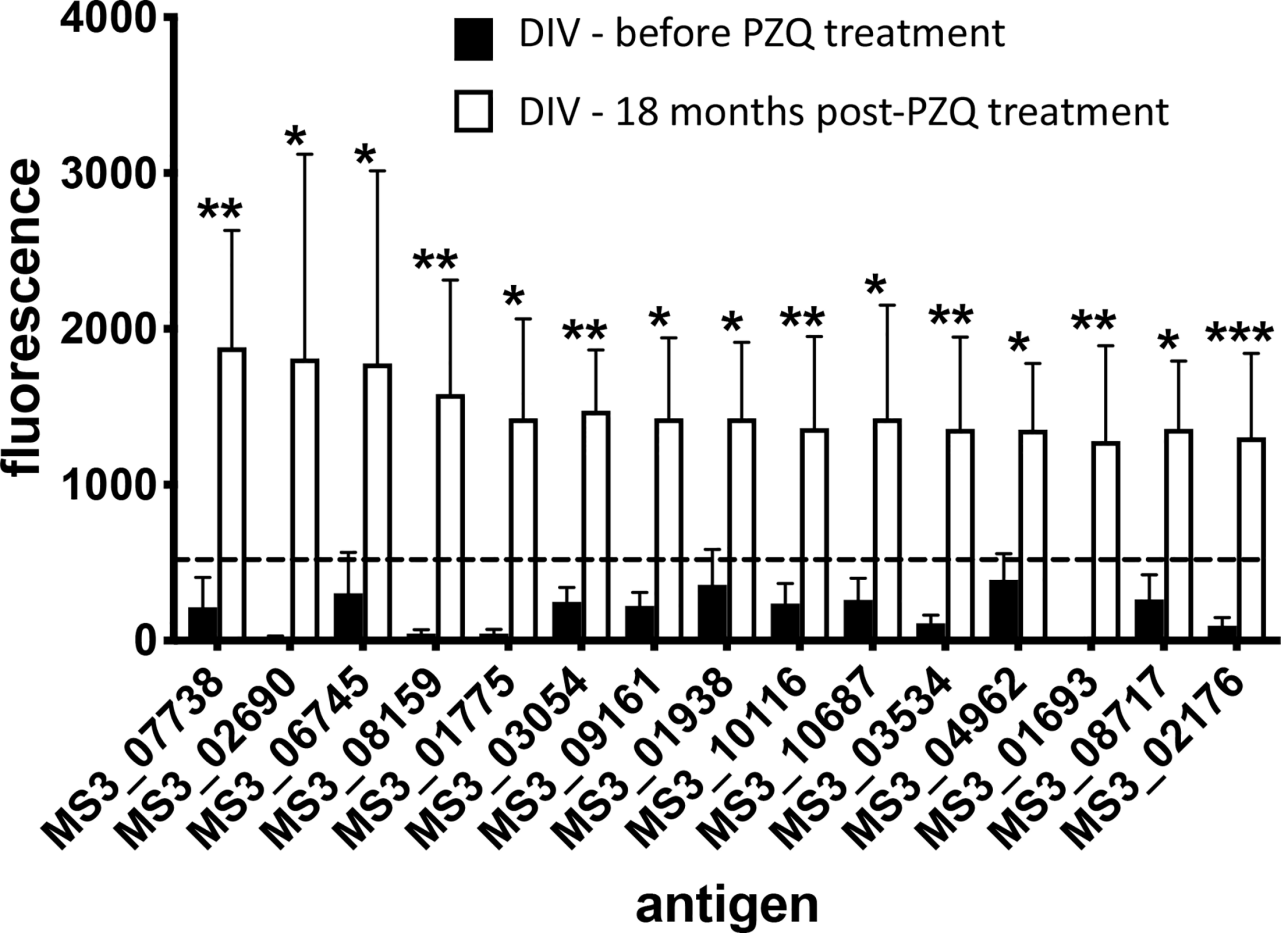
Antibody signatures to arrayed antigens differ in *S. haematobium*-infected subjects before and after PZQ treatment. Graph showing antigens which are targets of the top 15 significant (sorted by signal intensity) IgG responses in the DIV cohort before and after PZQ treatment. The dashed line represents the reactivity cut-off, determined as the mean SI + 1.5SD of the IgG response to all negative control (empty vector) spots. Significance was determined using a Mann-Whitney test. *P ≤ 0.05, **P ≤ 0.01, ***P ≤ 0.001.

A total of 138 proteins were targets of significantly (P<0.05) elevated SIs (IgG responses) in DIV subjects 18 months after PZQ treatment compared to baseline (**Table S2** and **Figure 2**). Fold-changes ranged from 2-3,210 and reactive proteins were sourced from various tissue proteomes (**Figure S1**; adult ES, extracellular vesicle, tegument, and soluble egg antigen) as well as from bioinformatic predictions. The top 15 antigens ranked by SI after PZQ treatment are provided in **Table 1** and **Figure 3**. Some proteins were immunoreactive at baseline but were the targets of elevated responses after PZQ, including the microexon gene family member MEG-8 (9-fold increase in SI) and cathepsin B (5-fold increase), whereas others were not recognized or recognized weakly at baseline but then strongly after PZQ treatment, such as synaptotagmin (130-fold increase) and trematode eggshell synthesis domain containing protein (38-fold increase). Only one protein, cystatin (MS3_01693), was completely unrecognized by IgG at baseline (SI = 0) but strongly recognized after PZQ treatment.

**Table 1:** Top 15 antigens which are the targets of significantly higher IgG responses in DIV individuals 18 months after, compared to before PZQ treatment.

Antigen (WBPS14^1^ Accession)	Description	SI before treatment^2^	SI after treatment^3^	Fold change^4^	*P* value^6^	Selection method for array inclusion^7^
MS3_07738	MEG-8 family	213.839	1995.197	9.330	0.004	bioinformatic
MS3_02690	synaptotagmin	14.729	1916.206	130.096	0.039	proteomic (T)
MS3_06745	eukaryotic translation elongation factor 1 beta 2	304.375	1874.503	6.159	0.014	proteomic (AES)
MS3_08159	trematode eggshell synthesis domain containing protein	44.755	1687.919	37.714	0.010	bioinformatic
MS3_01775	putative DNA-binding protein	45.404	1520.667	33.492	0.013	bioinformatic
MS3_03054	chaperonin containing TCP1, subunit 5 (epsilon)	247.255	1516.008	6.131	0.003	proteomic (SEA)
MS3_09161	thymidylate kinase	221.841	1492.275	6.727	0.023	proteomic (AES)
MS3_01938	proliferating cell nuclear antigen	358.357	1445.483	4.034	0.012	bioinformatic
MS3_10116	60S ribosomal protein L23	238.344	1427.153	5.988	0.007	bioinformatic
MS3_10687	cathepsin B-like cysteine proteinase	261.065	1398.814	5.358	0.042	bioinformatic/EV homologue
MS3_03534	UTP–glucose-1-phosphate uridylyltransferase	110.807	1380.553	12.459	0.006	proteomic (SEA)
MS3_04962	interferon-related developmental regulator 1	389.531	1365.497	3.505	0.042	bioinformatic
MS3_01693	cystatin	0.000	1365.483	n/a^5^	0.007	bioinformatic
MS3_08717	NADH dehydrogenase 1 alpha subcomplex subunit 13	264.685	1363.492	5.151	0.014	bioinformatic
MS3_02176	microsomal glutathione S-transferase 3	94.896	1362.997	14.363	0.001	proteomic (T)

^1^WormBase ParaSite online database, version 14 https://parasite.wormbase.org/Schistosoma_haematobium_prjna78265/Info/Index/.

^2^mean signal intensity of DV cohort before PZQ treatment.

^3^mean signal intensity of DIV cohort 18 months after PZQ treatment.

^4^fold change in mean signal intensity of DIV cohort 18 months after PZQ treatment compared to DIV cohort before treatment.

^5^n/a = not assessed; fold change unable to be calculated due to mean signal intensity of CI cohort below the reactivity threshold.

^6^significance of difference in mean signal intensity of DIV cohort 18 months after PZQ treatment compared to DIV cohort before treatment.

^7^proteins for array inclusion selected from bioinformatic analysis of second-generation S. mansoni array (16) or analysis of S. haematobium proteomes (13, 14).

T, S. haematobium adult tegument, AES, S. haematobium adult excretory/secretory products, SEA, S. haematobium soluble egg antigen.

**Figure 4:**
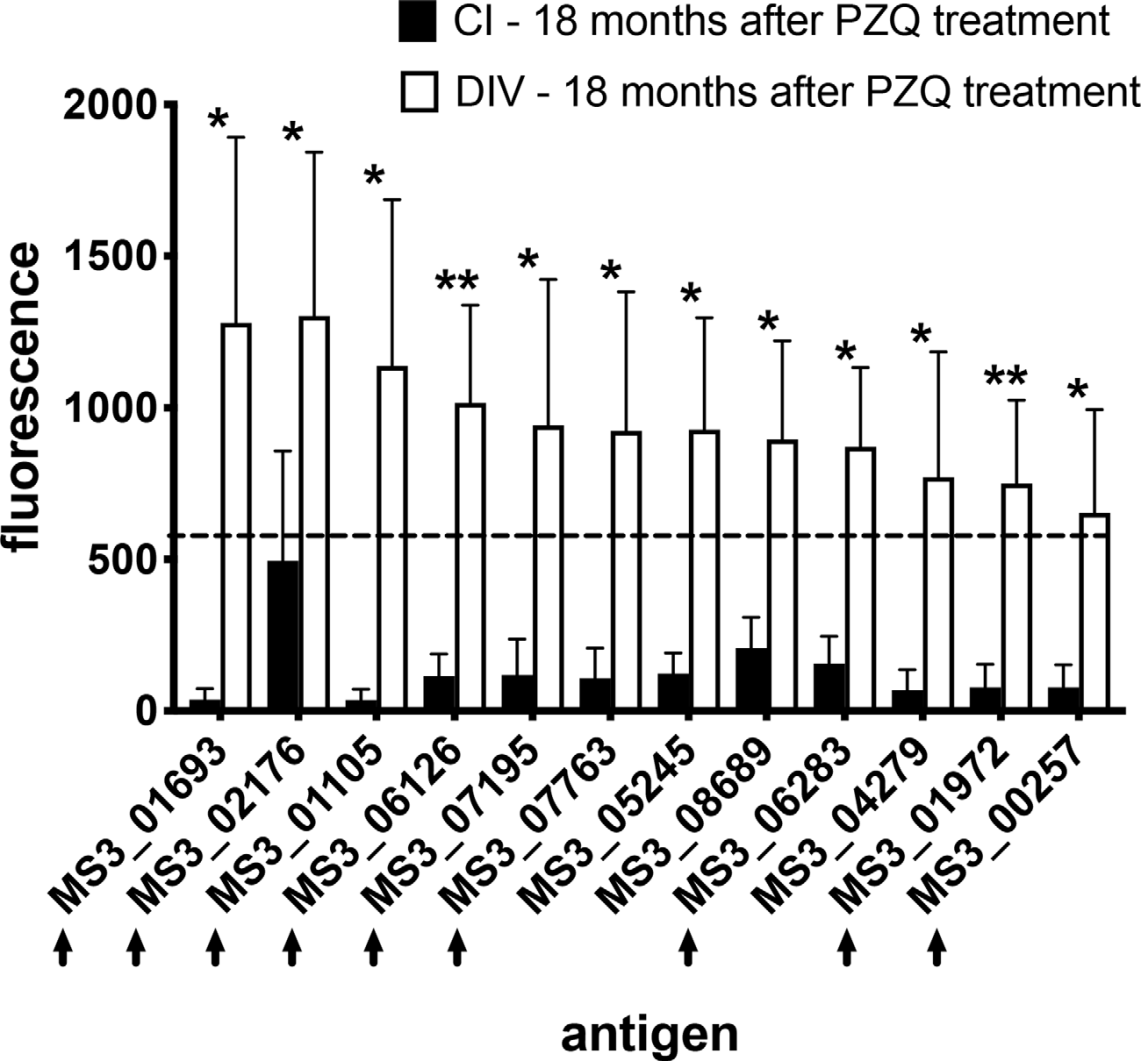
IgG profiles to arrayed antigens differ between *S. haematobium*-infected subjects who do and do not acquire resistance 18 months after PZQ treatment. Average signal intensities depicting IgG responses to each antigen are shown for the CI and DIV cohorts after PZQ treatment. Black arrows denote antigens which are also the target of significantly higher IgG responses in the DIV cohort after PZQ treatment. The dashed line represents the reactivity cut-off, determined as the average + 3SD of the IgG response to all arrayed antigens from the non-endemic negative cohort. Significance determined by student’s t-test. *P ≤ 0.05, **P ≤ 0.01.

We also compared immunoreactive proteomes between DIV and CI subjects 18 months after PZQ treatment (**Figure 2**). Twelve proteins were significantly more reactive in the DIV cohort after PZQ treatment, 9 of which were also significantly more reactive in the DIV cohort at 18 months post-treatment compared to the CI cohort 18 months post-treatment (**Table 2** and **Figure 4**). Cystatin (MS3_01693) was the target of the greatest fold-change (37-fold increase; *P*=0.030) in IgG reactivity after PZQ treatment. The percent identity across overlapping regions between *S. haematobium* and *S. mansoni* cystatins is 86%. *S. haematobium* cystatin contains a 12 amino acid predicted insertion that is not present in either *S. mansoni* or *S. japonicum* cystatins (**Figure S2**).

**Table 2:** Antigens which are the targets of significantly higher IgG responses in DIV, compared to CI, subjects 18 months after PZQ treatment.

Antigen (WBPS14^1^ Accession)	Description	CI cohort SI^2^	DIV cohort SI^3^	Fold change^4^	*P* value^5^	Selection method for array inclusion^6^
MS3_01693	cystatin	36.769	1365.483	37.137	0.030	bioinformatic
MS3_02176	microsomal glutathione S-transferase 3	495.452	1362.997	2.751	0.027	proteomic (T)
MS3_01105	universal stress protein	35.769	1214.122	33.943	0.030	proteomic (AES)
MS3_06126	sulfide quinone reductase, putative	114.734	1035.405	9.024	0.002	bioinformatic
MS3_07195	ribosomal protein S11	118.385	1000.316	8.450	0.026	bioinformatic
MS3_07763	succinyl-CoA ligase subunit beta, mitochondrial	107.417	958.094	8.919	0.045	proteomic (SEA)
MS3_05425	14 kDa subunit splicing factor 3b	123.558	934.925	7.567	0.050	proteomic (AES)
MS3_08689	synaptic vesicle membrane protein VAT-1-like protein	206.939	918.716	4.440	0.046	bioinformatic
MS3_06283	dexamethasone-induced Ras-related protein 1	156.625	875.308	5.589	0.024	bioinformatic
MS3_04279	saposin B domain-containing protein	68.077	822.758	12.086	0.047	bioinformatic/EV homologue
MS3_01972	leptin receptor overlapping transcript-like 1	77.240	768.733	9.952	0.004	proteomic (SEA)
MS3_00257	regulator of microtubule dynamics protein 1	77.878	697.027	8.950	0.045	bioinformatic

^1^WormBase ParaSite online database, version 14 https://parasite.wormbase.org/Schistosoma_haematobium_prjna78265/Info/Index/.

^2^mean signal intensity of CI cohort 18 month after PZQ treatment.

^3^mean signal intensity of DIV cohort 18 months after PZQ treatment.

^4^fold change in mean signal intensity of CI cohort compared to DIV cohort 18 months after PZQ treatment.

^5^significance of difference in mean signal intensity of CI cohort compared to DIV cohort 18 months after PZQ treatment.

^6^proteins for array inclusion selected from bioinformatic analysis of second-generation S. mansoni array (16) or analysis of S. haematobium proteomes (13, 14).

T, S. haematobium adult tegument, AES, S. haematobium adult excretory/secretory products, SEA, S. haematobium soluble egg antigen.

### Vaccination of Mice With Recombinant *S. mansoni* Cystatin Significantly Reduces Egg Output in a *S. mansoni* Challenge Model

**Figure 5:**
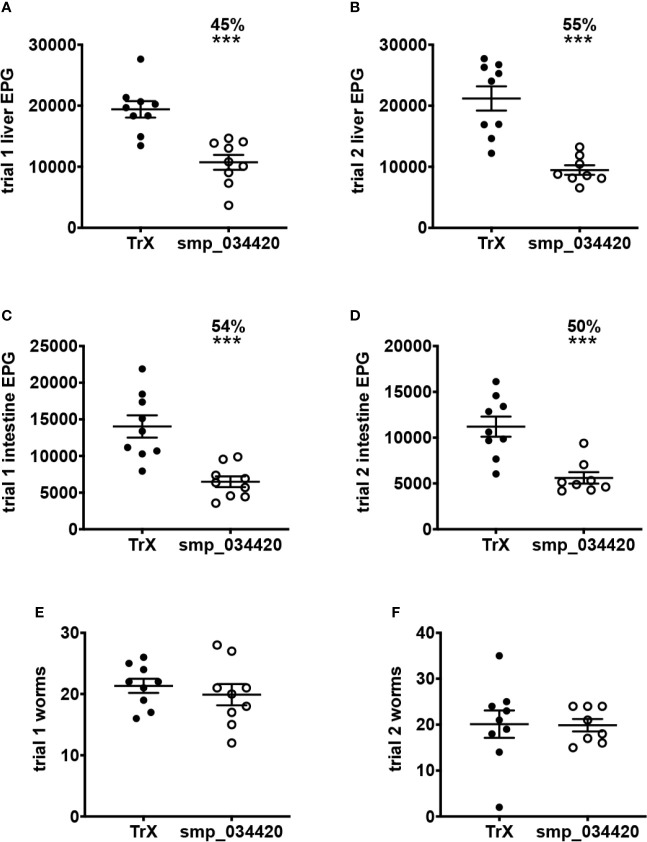
Vaccination of mice with recombinant *S. mansoni* cystatin confers protection in the form of significantly reduced liver and intestinal *S. mansoni* egg burdens but not adult fluke burdens across two independent trials. **(A)** liver egg reduction trial 1, **(B)** liver egg reduction trial 2, **(C)** intestinal egg reduction trial 1 and **(D)** intestinal egg reduction trial 2. The percentage of reductions in parasite burden are above each dataset. Differences between cystatin (smp_034420) vaccinated group and the control group vaccinated with recombinant thioredoxin (TrX) were analysed with a student’s t-test. ***P < 0.001. Adult fluke burdens did not significantly differ between vaccinated and control groups in either trial **(E, F)**.

The mouse model of *S. haematobium* is sub-optimal, with large numbers of challenge cercariae required to get relatively low numbers of adult flukes. Moreover, unlike in humans, adult *S. haematobium* do not reach the urogenital vasculature in mice, and instead are usually found in the mesenteries in the liver and bowel. We therefore identified the *S. mansoni* orthologue of *S. haematobium* cystatin (Smp_034420; **Figure S2**), expressed it in recombinant form, and tested its vaccine efficacy in a mouse model of *S. mansoni* infection. Recombinant *S. mansoni* cystatin was insoluble in the absence of chaotropic agents and was therefore solubilized with 8M urea and purified by nickel-NTA chromatography under denaturing conditions (not shown). In two independent trials, vaccination of mice with *S. mansoni* cystatin was immunogenic (**Figure S3**). Significant reductions in liver egg burdens (45-55%) and intestinal egg burdens (50-54%) were observed in mice vaccinated with cystatin compared to control mice vaccinated with *E. coli* thioredoxin in both trials (**Figures 5A–D**). Vaccination with cystatin did not, however, result in a significant difference in adult fluke burdens in either trial (**Figures 5E, F**). Trial 1 liver eggs: TRX group 19,404 ± SD 4,052 *vs.* cystatin 10,730 ± 3,652, *P* = 0.0001; trial 2 liver eggs: TRX group 21,191 ± 5,959 *vs.* cystatin 9,466 ± 2,215, *P* = 0.0001). Trial 1 intestinal eggs: TRX group 14,029 ± 4,530 *vs.* cystatin 6,484 ± 2,206, *P* = 0.0002; trial 2 intestinal eggs: TRX group 11,204 ± 3,295 *vs.* cystatin 5,609 ± 1,769, *P* = 0.0003.

## Discussion

PZQ is widely used to treat human schistosome infections and has two main effects on adult flukes - paralysis and tegument damage. Experimental work in mice has demonstrated that PZQ treatment exposes tegumental antigens (22, 23), and studies in disease-endemic areas have shown that repeated rounds of PZQ therapy results in enhanced Th2 cytokine and antibody responses, notably IgE against surface membrane proteins (7, 24). In addition, adult worms and eggs suppress schistosome-specific immune responses (25) so that worm death results in increased responsiveness to schistosome antigens.

Only limited research has been conducted on the nature of DIV, particularly in the post-genomic era. As such, we exploited various omics datasets to generate the first *S. haematobium* proteome array and probed the 1,000 proteins with sera from DIV and CI individuals to identify antigens that are selective targets of elevated IgG responses in human subjects after PZQ therapy. The major target antigens of the DIV response included a range of secreted, membrane and intracellular proteins sourced from ES products, EVs, tegument and eggs. While numerous candidate antigens were the targets of significantly elevated serum antibody responses after PZQ therapy, we focused on one in particular, MS3_01693, a member of the cystatin family of cysteine protease inhibitors. Our rationale for choosing cystatin was multi-factorial, including (i) elevated antibody titers in DIV cohort after PZQ treatment compared to CI group, including a complete absence of detectable antibodies in DIV subjects pre-treatment (**Tables 1** and **2**); (ii) absence of existing literature on cystatin vaccines in flukes but evidence of a protective role for anti-cystatin antibodies against parasitic nematodes (26, 27) and blood-feeding ticks (28); (iii) proven role of the *Schistosoma japonicum* orthologue in suppressing inflammation.

While not previously reported as anti-schistosome vaccine antigens, a recombinant form of cystatin from *S. japonicum* has immunomodulatory properties and confers protection against a range of inducible inflammatory diseases in mice (29–32), possibly *via* inhibition of antigen presenting cell lysosomal cysteine protease, thereby interrupting antigen processing and presentation (33) and macrophage polarization (34). It is feasible that *S. haematobium* cystatin performs a similar immunoregulatory role in urogenital schistosomiasis, and that vaccine-induced antibodies interrupt its ability to attenuate inflammation and the development of an anti-schistosome protective immune response. Indeed, a multivalent subunit vaccine against a blood-feeding gastrointestinal nematode parasite of sheep confers upwards of 70% protection against challenge infection and contains numerous immunomodulatory ES proteins (35).

A number of other antigens were identified as selective targets of DIV IgG responses, including MS3_02176, a microsomal glutathione S-transferase 3 (GST-3). Microsomal GSTs are membrane -associated proteins primarily involved in the production of eicosanoids (36), not to be mistaken with cytosolic GSTs including the *S. mansoni* 28 kDa GST vaccine that progressed into late phase clinical trials (37) but is no longer in clinical development. Little is known about the role of this family of proteins in parasitic helminths, but given the importance of leukotrienes and other eicosanoid inflammatory mediators, this family warrants further attention and should be the focus of future vaccine trials in animal models.

Antibodies targeting MS3_04279, a member of the saposin family of pore-forming proteins were 12-fold greater in DIV than CI subjects post-PZQ treatment. Saposins are known to be immunogenic in *S. mansoni* infection (38) and are candidate serodiagnostic antigens in *S. japonicum* infection (39). Recombinant saposins from the liver flukes *Fasciola hepatica* and *Fasciola gigantica* confer protection against challenge infection in animal models (40, 41). Saposins from the gastrodermis of *S. mansoni* were tested as subunit vaccines in the mouse model and did not confer protection (38), but based on these findings herein, the *S. haematobium* proteins should be tested before final down-selection.

The protein that was the target of the highest-fold change in DIV IgG responses before and after treatment was synaptotagmin. The role of this family of proteins has not been addressed in parasitic helminths, but in the free-living nematode, *Caenorhabditis elegans*, synaptotagmin-1 functions as a calcium-sensing protein and orchestrates the membrane association/disassociation cycle of Rab3 in the recruitment of synaptic vesicles (42). Synaptotagmin was identified in the tegument proteome of *S. haematobium* (14), and an important role for this protein has been described in presynaptic exosome release in *Drosophila* (43). It is feasible that like other proteins on the surface of schistosome EVs (44), antibodies to synaptotagmin might interrupt fluke vesicle uptake by host vascular endothelial and immune cells, thereby interrupting an important mechanism of immunoregulation. It is also possible that synaptotagmin might be released by dying worms (after PZQ treatment), thereby driving an immune response that is not meaningful in terms of protection, and further work is required to shed light on this issue.

Herein we focused our schistosomiasis vaccine antigen discovery efforts on the targets of IgG responses. While IgE has been shown to be an important antibody isotype in the acquisition of DIV (45), anti-helminth vaccines that induce IgE pose serious risks for development of serious adverse events, including allergic reactions (46) and possibly even anaphylaxis. Current candidate schistosomiasis vaccines confer protection in animal models and are targets of IgG but not IgE responses in exposed individuals (18, 47), so the candidate antigens identified in this study now require screening with sera from infected subjects to assess their IgE reactivity.

Crosnier and colleagues recently described the expression of 115 *S. mansoni* proteins and screened them for immunoreactivity with sera from human subjects who were experimentally infected with male parasites (48). However, to express 1,000 proteins in mammalian cells would require a Herculean effort and substantial funding and definitely is not considered high-throughput. The beauty of the system we used is the high-throughput nature of the immunomics approach where crude cell-free reverse transcription-translation products are printed and probed. While some conformational epitopes might not be faithfully reproduced in this system, the ease with which large numbers of proteins can be rapidly produced and screened provides sufficient support for such an approach.

In summary, we used an immunomics based approach to screen 1,000 *S. haematobium* proteins to identify those that are selectively or preferentially recognised by IgG antibodies from human subjects who developed praziquantel-induced immunologic resistance to schistosomiasis. Multiple candidate antigens were identified, and one, cystatin, was validated as a possible anti-fecundity and potentially transmission-blocking vaccine by reducing egg output from vaccinated individuals. Much work remains to further assess different recombinant production systems, formulations and delivery modes of cystatin and other promising antigens, including testing their efficacy in a chemotherapy-linked delivery regimen in a robust animal model of *S. haematobium* infection, such as non-human primates.

## Data Availability Statement

The datasets presented in this study can be found in online repositories. The names of the repository/repositories and accession number(s) can be found in the article/**Supplementary Material**.

## Ethics Statement

The studies involving human participants were reviewed and approved by Medical Research Council of Zimbabwe. Written informed consent to participate in this study was provided by the participants’ legal guardian/next of kin. The animal study was reviewed and approved by James Cook University Animal Ethics Committee.

## Author Contributions

MP, FM, PF, and AL conceived the study design. MP, BT, LB, RN, AJ, and JS carried out the work. MP, RN, JS, PF, and AL analysed the data. TM and FM provided clinical samples. AL and MSP wrote the manuscript. All authors contributed to the article and approved the submitted version.

## Funding

This study received financial support from Merck KGaA, Darmstadt, Germany, and the Australian Trade and Investment Commission (Australian Tropical Medicine Commercialisation grants program ATMC50322). The funder was not involved in the study design, collection, analysis, interpretation of data, the writing of this article or the decision to submit it for publication. AL was funded by an NHMRC Senior Principal Research Fellowship (APP1117504). FM was funded by the Thrasher Research Fund (12440) and the Wellcome Trust (108061/Z/15/Z). The authors thank Atik Susianto for maintenance of the parasites and laboratory animals. They also gratefully acknowledge the NIAID Schistosomiasis Research Center of the Biomedical Research Institute, Rockville, MD, USA for the provision of *S. mansoni*-infected *B. glabrata* snails for this work through NIH-NIAID contract HHSN2722017000141 for distribution through BEI resources.

## Conflict of Interest

BG was employed by Ares Trading, S.A.

The remaining authors declare that the research was conducted in the absence of any commercial or financial relationships that could be construed as a potential conflict of interest.

## Supplementary Material

Supplementary Figure 1 Heatmap showing source of *Schistosoma haematobium* antigen targets (rows) of significantly elevated IgG responses in individual DIV subjects (columns) before (S0) and after (S3) praziquantel treatment. AES, Adult Excretory/Secretory proteome; EV, Extracellular Vesicle proteome; T, Tegument proteome; SEA, Soluble Egg Antigen proteome; Sm, bioinformatically identified homolog/ortholog of *S. mansoni* protein predicted to be secreted.

Supplementary Figure 2 Amino acid alignment of *Schistosoma. haematobium* (Sh), *S. japonicum* (Sj) and *S. mansoni* (Sm) cystatins.

Supplementary Figure 3 ELISA showing serum IgG response of mice vaccinated with recombinant *Schistosoma mansoni* cystatin (smp_034420; trial 1) prior to immunisation and post-immunisation but prior to challenge infection.
