# Supplementary figures and images for: Immunomics-Guided Antigen Discovery for Praziquantel-Induced Vaccination in Urogenital Human Schistosomiasis
Source: Front Immunol. 2021 May 25;12:663041. doi: 10.3389/fimmu.2021.663041 (PMC8186320; doi:10.3389/fimmu.2021.663041)

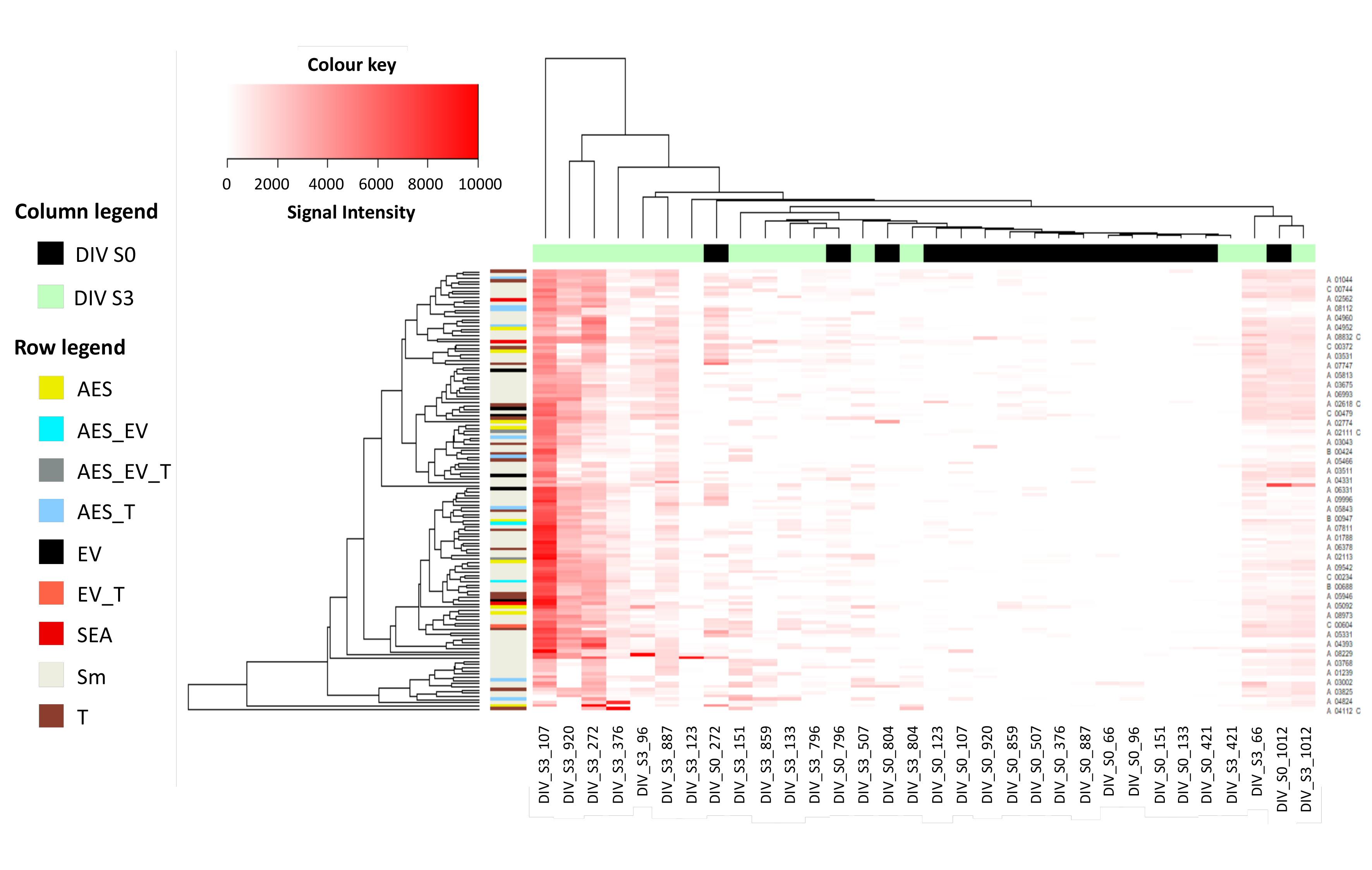

Supplement: Supplementary Figure 1 — Heatmap showing source of Schistosoma haematobium antigen targets (rows) of significantly elevated IgG responses in individual DIV subjects (columns) before (S0) and after (S3) praziquantel treatment. AES, Adult Excretory/Secretory proteome; EV, Extracellular Vesicle proteome; T, Tegument proteome; SEA, Soluble Egg Antigen proteome; Sm, bioinformatically identified homolog/ortholog of S. mansoni protein predicted to be secreted. [file Image_1.tif]

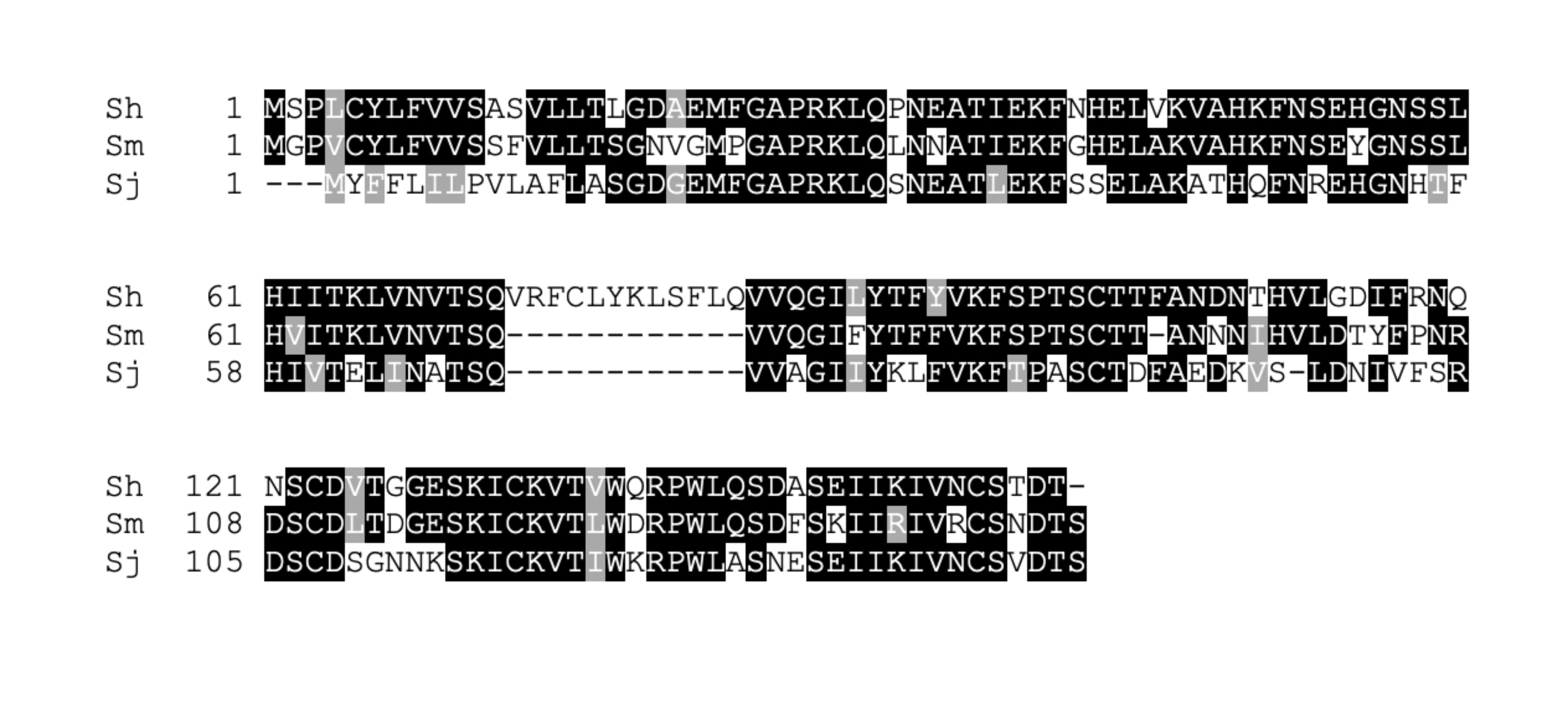

Supplement: Supplementary Figure 2 — Amino acid alignment of Schistosoma. haematobium (Sh), S. japonicum (Sj) and S. mansoni (Sm) cystatins. [file Image_2.tif]

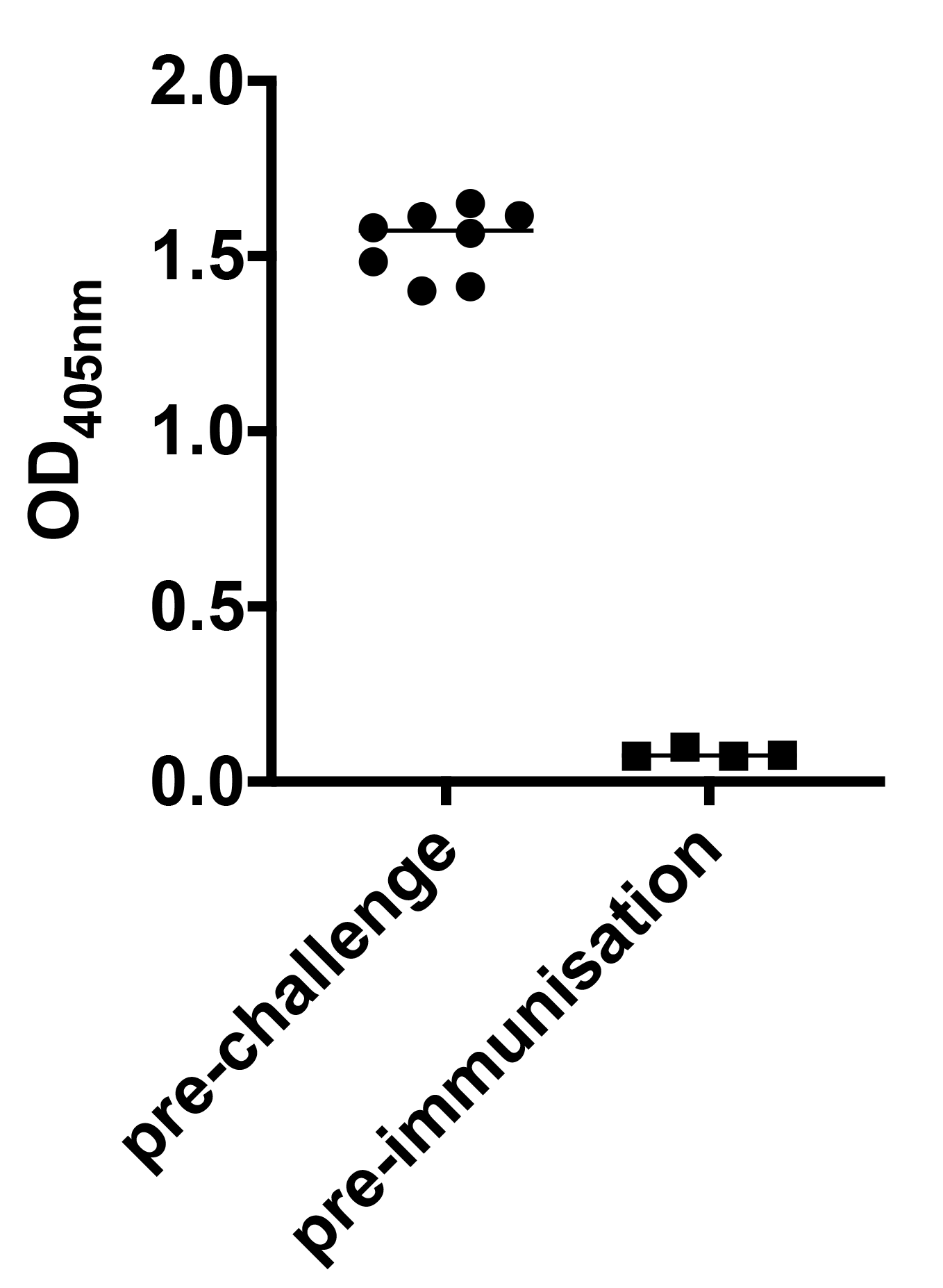

Supplement: Supplementary Figure 3 — ELISA showing serum IgG response of mice vaccinated with recombinant Schistosoma mansoni cystatin (smp_034420; trial 1) prior to immunisation and post-immunisation but prior to challenge infection. [file Image_3.tif]
